# Phosphorus Fertilization Enhances Productivity of Forage Corn (*Zea mays* L.) Irrigated with Saline Water

**DOI:** 10.3390/plants10122608

**Published:** 2021-11-28

**Authors:** Hamza Bouras, Ahmed Bouaziz, Redouane Choukr-Allah, Abdelaziz Hirich, Krishna Prasad Devkota, Bassou Bouazzama

**Affiliations:** 1 Department of Crop Production, Protection and Biotechnology, Hassan II Institute of Agronomy and Veterinary Medicine, Rabat 10101, Morocco; bourashamza07@gmail.com (H.B.); hmadbouaziz@gmail.com (A.B.); redouane53@yahoo.fr (R.C.-A.); 2 African Sustainable Agriculture Research Institute (ASARI), Mohammed VI Polytechnic University (UM6P), Laayoune 70000, Morocco; krishna.devkota@um6p.ma; 3 National Institute for Agricultural Research (INRA), Beni Mellal 23020, Morocco; bassoubouazzama@gmail.com

**Keywords:** irrigation, biomass yield, stomatal conductance, silage, best practices

## Abstract

Salinity is a major problem affecting crop production in many regions in the world including Morocco. Agricultural practices such as fertilization could be useful to overcome this problem and improve crop productivity. The objective of our study was to evaluate the combined effect of phosphorus fertilization and irrigation water salinity on growth, yield, and stomatal conductance of forage corn (*Zea mays* L.) cv. “Sy sincerro”. Field experiments were carried out for two years testing four levels of irrigation water salinity (ECw = 0.7; 2, 4, and 6 dS·m^−1^) and three rates of phosphorus (105, 126, and 150 kg P_2_O_5_·ha^−1^) fertilization conducted in a split-plot design with three replications. The obtained results show that irrigation water salinity had a negative effect on all monitored parameters. For instance, the dry matter yield reduced by an average of 19.3 and 25.1% compared to the control under saline irrigation with an EC value equal to 4 and 6 dS·m^−1^, respectively. The finding also showed that phosphorus applications tend to increase root weight, root length, stem length, leaf stomatal conductance, grain yield and dry matter yield under salinity conditions. For example, the addition of phosphorus with a rate of 126 and 150 kg P_2_O_5_·ha^−1^ respectively improved dry matter yield by an average of 4 and 9% under low salinity level (ECw = 2 dS·m^−1^), by 4 and 15% under medium salinity (4 dS·m^−1^), and by 6 and 8% under a high salinity level (6 dS·m^−1^). Our finding suggests that supplementary P application could be one of the best practices to reduce the adverse effects of high salinity on growth and development of forage corn.

## 1. Introduction

Salinity is a major constraint that limits crop production in many regions of the world. It is estimated that approximately 20% of cultivated land in the world and 33% of irrigated land, are salt-affected and degraded [1]. Soil salinity is increasing in many parts of the world and has become a serious economic and environmental constraint limiting agricultural productivity and profitability, reducing water and nutrient-use efficiencies, and causing land abandonment and desertification. It has been predicted that more than 50% of the arable land could be salinized by the year 2050 [2]. Salinization is the main cause of soil degradation in irrigated areas induced by several factors: arid climate, use of saline water for irrigation, poor drainage system, over-irrigation, and misuse of fertilizers and phytosanitary products.

Soil salinity is a major problem in the Mediterranean region where 27.3 million ha of soil have been salt-affected with 7.3 million ha in Morocco, Spain, Tunisia, and Turkey [3]. According to the Moroccan Department of Agriculture, soil salinization affects nearly 500,000 ha and causes significant losses in agricultural productivity. Secondary salinization, which is the fastest form of soil degradation in irrigated areas, affects around 160,000 ha or around 16% of irrigated land in Morocco [4]. The Tadla is one of the main irrigated salt-affected agricultural zones in Morocco, with a total arable land equal to 259,600 ha, where 49% is irrigated. According to ORMVAT [5], water salinity is highly variable around an overall average of 2.76 dS·m^−1^ in the perimeter of Tadla. The highest electrical conductivity (EC) is found at Beni Amir perimeter with a maximum value of 8.4 dS·m^−1^.

Salinity is a major abiotic stress that limits the growth and productivity of a wide variety of crop species across the world [6,7,8]. Increased salinity leads to a reduction in the plant biomass, leaf area, stem, and root length and ultimately crop yield [9]. Salinity causes three major stresses on the plant growth: (a) it increases osmotic pressure in the soil solution, which causes reduced water availability; (b) high concentration of toxic ions, especially sodium (Na+) and chloride (Cl-) in the soil solution that leads to increased accumulation of these ions in leaves; (c) it causes nutrient disorder and deficiency in plants [10]. Under saline conditions, plants cannot tolerate the toxic amounts of solutes in their cytoplasm. They either restrict a large amount of solutes in their vacuole or compartmentalize these toxic ions in tissues to support their normal metabolic activities [11]. Salt tolerance of a plant is controlled by several factors including soil, water, plant, and environmental conditions [12]. There are several mechanisms and strategies that enable plants to survive under salinity conditions including: ion vacuolation, accumulation of adaptive osmolytes, osmotic adaptation and adjustment, selective transport and uptake of ions, salt exclusion, ion homeostasis, and salt excretion in plant organs such as leaves [13].

Corn (*Zea mays* L.) is one of the three most important cereal crops (after wheat and rice) that can be grown in a wide range of climates [14]. Several early studies have evaluated the impact of salinity on maize, but only a few have reported the interactive effect of salinity and fertilization. For instance, Rhoades et al. [15] reported that maize yield reduced by 50% under soil salinity with an EC value of 5.9 dS·m^−1^. They also reported that the salt tolerance threshold value of maize (beyond which yield reduction occurs) is 1.8 dS·m^−1^ and maize is moderately sensitive to salinity. Other studies also found similar results, e.g., maize yield reduction by 0% at ECe 1.7 dS·m^−1^, 10% at 2.5 dS·m^−1^, 25% at 3.8 dS·m^−1^, 50% at 6 dS·m^−1^, and 100% at ECe 10 dS·m^−1^ [16]. Irrigation with water salinity below 3 g·L^−1^ reduced maize yield by 10% compared with freshwater irrigation [17]. However, in the long-term even with the low concentrations of salt, a significant yield loss might occur due to salt accumulation of salt in the root zone. The annual average yield and crop water productivity of spring maize decreased by 5.3 and 2.6%, respectively under saline irrigation water compared to freshwater irrigation [18].

The fertilizers supplied through irrigation water (fertigation) can reduce soil salinization and mitigate salinity stress impacts as it improves fertilizer use efficiency and nutrient availability [19]. In drip irrigation with fertilizer, the timing of application, fertilizer’s concentration and rate can be easily controlled [1]. Phosphorus (P) is the primary nutrient that restricts growth and development and ameliorates salt-induced reduction in crop yield [20]. Phosphorus fertilization in salt-affected soils improved crop growth and yield in 34 out of 37 crops studied, but did not increase the salt tolerance of crops [21]. Phosphate fertilization under saline conditions was shown to increase crop productivity. Such a beneficial effect generally involves a positive phosphorus-salinity interaction, especially when the salinity is moderate [21].

Phosphorus is a primary nutrient necessary for plant growth and development involved in many metabolic processes including energy transfer, signal transduction, biosynthesis of macromolecules, photosynthesis, and respiration [22]. Increasing P rate leads to increased salt tolerance of various crops such as sesame [23], tomato [24], micro-propagated potato [25], spinach [26], rice [27], pepper seedling [28], pistachio seedlings [29], barley [30], wheat [31], cucumber [32], wild (*Hordeum maritimum*) and cultivated barley (*Hordeum vulgare*) [33], common bean [34], green beans [35], and sugar beet [36]. Belouchrani et al. [37] reported that phosphorus application resulted in a remarkable improvement of sorghum growth and tolerance towards salinity, where it increased plant height, dry matter yield, nitrogen and phosphorus uptake, and accumulation of osmolytes such as proline. Malik et al. [38] reported that there is a synergistic relationship between phosphorus and other beneficial elements such as K^+^, Ca^2+^ and Mg^2+^, which stimulated an osmotic effect and therefore resulted in salt tolerance to some extent. Hence, the main objective of this study was to evaluate the interaction of phosphorus supply with different salinity of irrigation water and its effect on growth and productivity of forage corn (*Zea mays* L.) cv. “Sy Sincerro”. 

## 2. Results

### 2.1. Analysis of Variance of Growth and Physiological Parameters

Table 1 summarizes the results of the ANOVA (analysis of variance) of measured growth and physiological parameters as affected by irrigation water salinity and P rates during 2019–2020. Salinity affected (*p* < 0.05) all parameters in both years except root fresh weight in both years. Phosphorus fertilization also affected (*p* < 0.05) most of the parameters except leaf number, plant height and leaf area in both years. The interaction effect was significant for shoot fresh weight and plant dry weight in 2019 and for plant dry weight, dry matter yield and grain yield in 2020.

### 2.2. Growth Parameters

Monitored plant growth parameters for both seasons are presented in Table 2. Under high salinity level (6 dS·m^−1^) number of leaves, plant height, root fresh weight, shoot fresh weight, and leaf area showed an average reduction by 12–26%. Conversely, root length increased by 16 and 12% under moderate (4 dS·m^−1^) and high salinity (6 dS·m^−1^), respectively (as an average of both seasons). Increasing P fertilization under salinity conditions improved all growth parameters (*p* ≤ 0.05); the highest values of most parameters were obtained when the plant was supplied with 150 kg P_2_O_5_·ha^−1^ of P fertilization.

### 2.3. Stomatal Conductance

**Figure 1:**
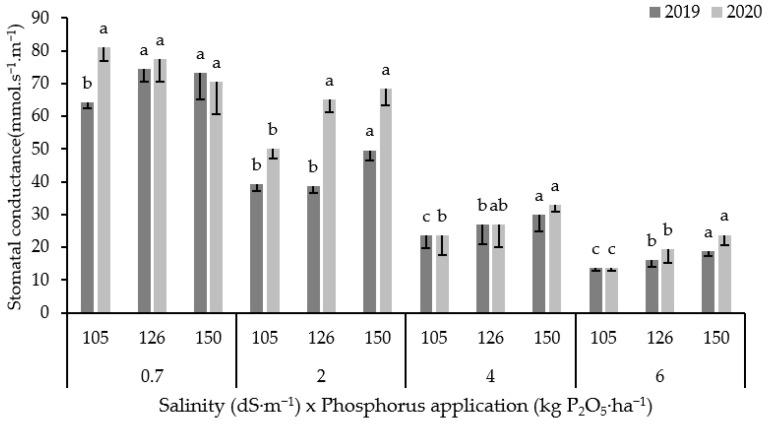
Variation in stomatal conductance for 2019 and 2020 seasons under different salinity levels and P rates. Error bars indicate the standard deviation. Phosphorus treatments under the same salinity level and for the same season without a common letter are significantly different at *p* < 0.05. Small letters (a, b and c) indicate the statistically homogenous groups.

The combined data over two years showed that salinity has a reduced stomatal conductance (gs) by 30, 63 and 77% under saline irrigation with EC values 2, 4 and 6 dS·m^−1^, respectively compared to control (0.7 dS·m^−1^) (Figure 1). It is obvious from the obtained results that increased P fertilization significantly improved gs under saline conditions for both seasons. For example, an application of 150 kg P_2_O_5_·ha^−1^ resulted in an average increase in gs by 32, 33 and 55% under 2, 4 and 6 dS·m^−1^, respectively compared to control. The results indicate that the gs improvement was greater under high salinity than under low salinity conditions.

### 2.4. Dry Matter and Seed Yield

**Figure 2:**
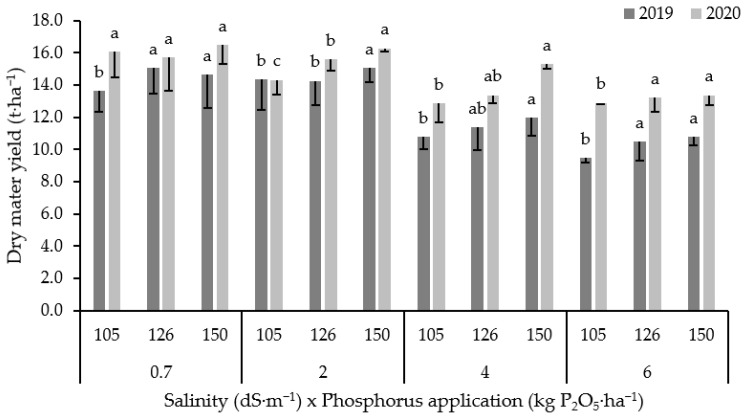
Variation in dry matter yield for both 2019 and 2020 seasons under different salinity levels and P rates. Error bars indicate the standard deviation. Phosphorus treatments under the same salinity level and for the same season without a common letter are significantly different at *p* < 0.05. Small letters (a, b and c) indicate the statistically homogenous groups.

Figure 2 illustrates the variation of dry matter yield influenced by both salinity and P fertilization rates. As expected, salinity decreased dry matter yield and the average reduction rate (over two seasons) was equal to 2, 17 and 23% compared to control under irrigation water salinity with an EC value equal to 2, 4 and 6 dS·m^−1^, respectively. However, the dry matter yield responded positively to the increased P rate, and this improvement was more pronounced under salinity conditions compared to freshwater irrigation conditions. For example, under low salinity level (2 dS·m^−1^) the application of 126 and 150 kg P_2_O_5_·ha^−1^ significantly increased dry matter yield by 4 and 9%, respectively (averaged data over two seasons). While under moderate salinity (4 dS·m^−1^), the increment rate was significant as it equaled to 4 and 15%, respectively. Conversely, the application of 126 and 150 kg P_2_O_5_·ha^−1^ increased dry matter yield by 4 and 5%, respectively under high level of salinity (EC 6 dS·m^−1^). The interaction between irrigation water salinity x P rate was significant only for 2020, which indicates that grain yield responded differently to phosphorus application under high salinity irrigation water. In fact, grain yield was not affected by phosphorus application under freshwater irrigation (0.7 dS·m^−1^); contrarily, it increased (*p* < 0.05) in high salinity level with increasing P rates.

### 2.5. Grain Yield

**Figure 3:**
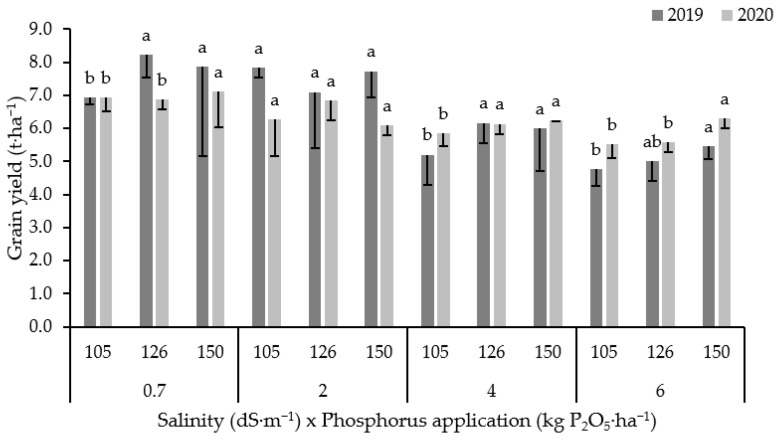
Variation in grain yield for both 2019 and 2020 seasons under different salinity levels and P rates. Error bars indicate the standard deviation. Phosphorus treatments under the same salinity level and for the same season without a common letter are significantly different at *p* < 0.05. Small letters (a, b and c) indicate the statistically homogenous groups.

Similarly to dry matter yield, grain yield of forage corn was significantly affected by salinity level and P rate (Figure 3). The interaction between salinity x P rate on grain yield was significant only for 2020 season. The results showed that grain yield responded differently to phosphorus application and was not affected by phosphorus application under low salinity level (2 dS·m^−1^) contrarily to other salinity levels where the phosphorus effect was significant. Salinity caused a reduction in the grain yield by 5, 19 and 26% (as an average of both seasons) compared to control under saline irrigation with an EC value equal to 2, 4 and 6 dS·m^−1^, respectively. However, P fertilization helped the plant withstand salinity and improved grain yield (*p* < 0.05), especially under moderate (4 dS·m^−1^) and high (6 dS·m^−1^) salinity conditions. While under low salinity (2 dS·m^−1^), grain yield was not affected (*p* > 0.05) by the P rate. Grain yield increment rate when the crop was exposed to a P application of 126 and 150 kg P_2_O_5_·ha^−1^ was 11% under moderate salinity (4 dS·m^−1^), and 3 and 15% under high salinity (6 dS·m^−1^), respectively.

### 2.6. Correlation Matrix

**Figure 4:**
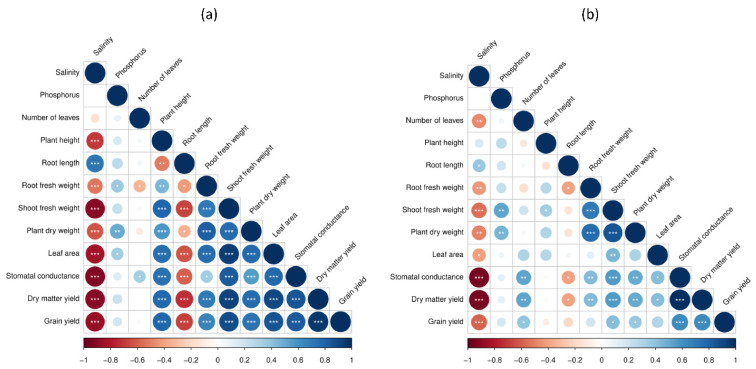
Pearson’s correlation matrix for all the investigated parameters during 2019 (**a**) and 2020 (**b**) *, **, *** indicates significance level at *p* = 0.05, 0.01, 0.001 respectively. Color gradient corresponds to the Pearson coefficient of correlation.

Pearson’s correlation analysis was conducted for the two growing seasons separately for all investigated agro-morphological parameters. The results obtained are shown in Figure 4. For the first trial season (Figure 4a), except for the root length parameter which had a significant positive correlation with salinity, a significant strong negative correlation was observed among most of the parameters and especially for shoot fresh weight, leaf area, stomatal conductance, dry matter yield, and grain yield. Results were confirmed during the second season (Figure 4b). The analysis also revealed a positive and moderate correlation of root, shoot and plant dry weight with the phosphorus application.

### 2.7. Principal Component Analysis (PCA)

**Figure 5:**
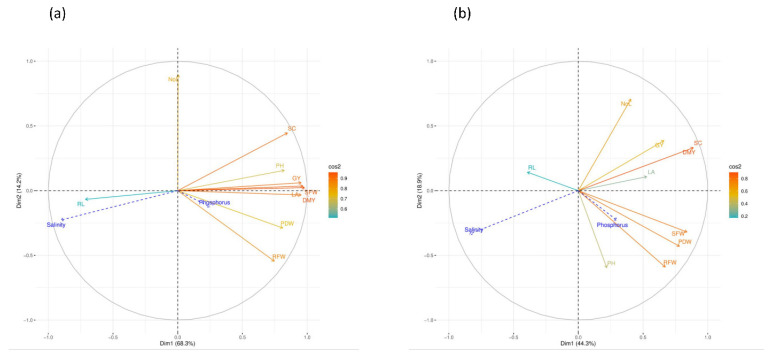
Correlation circle of variables on the principal two components for all investigated parameters during 2019 (**a**) and 2020 (**b**). NoL: Number of leaves, PH: Plant height, RL: Root length, RFW: Root fresh weight, SFW: Shoot fresh weight, PDW: Plant dry weight, LA: Leaf area, SC: Stomatal conductance, DMY: Dry matter yield, GY: Grain yield. Color gradient corresponds the quality of representation of the variables using the cos^2^ of its coordinates. Blue arrows show the projection of the supplementary dependent variables.

Figure 5 displays the correlation circle of the investigated variables for each cropping season. The quality of the representation of the variables was assessed by the squared cosine. Results of PCA indicate that the first two principal components represent 82.5% and 63.2% of the data variability respectively for the first (Figure 5a) and the second season (Figure 5b). PC1 axis was explained by shoot fresh weight, dry matter yield, stomatal conductance, and plant dry weight, while the number of leaves is the main variable that contributed to the formation of the PC 2 axis. The projection of supplementary dependent variables showed that the salinity was correlated negatively with all PC1 variables. Moreover, root fresh weight is positively correlated with the phosphorus rate applied.

## 3. Discussion

### 3.1. Effect of Salinity on Plant Growth and Yield

All growth and yield parameters of forage maize were affected in varying degrees depending on salinity level and applied phosphorous rate. Yield reduction under salinity is mainly explained by the reduction in photosynthetic activity. This study found that stomatal conductance consistently decreased when corn plants were exposed to high salinity (Figure 1). Soil salinity affects photosynthesis and thus the grain yield by reducing stomatal conductance [39]. The reduction in stomatal conductance reduces the photosynthetic rate and water uptake [40]. Salinity reduces photosynthetic activities in many ways, for example by inhibiting photosystem II [41], downregulating photosynthesis by stomatal closure, reducing photochemical and carbon metabolism [42], injuring chloroplast and stomata structure [43], and reducing chlorophyll and carotenoid content [44]. Our finding is consistent with previous findings in summer maize [45], bean and cotton [46], wheat [47], and grapevine [48], where soil salinity decreased photosynthetic rate, crop growth, and yield. 

The lower yield under saline conditions can be explained by the reduced plant height and leaf area (Table 2). Salinity stress reduces average grain weight and grain number per cob as it limits sink size and reduces the enzyme invertase activity [49]. The low yield under salinity stress might be due to the reduction of translocation of assimilates, which consequently causes poor grain filling [50]. Moreover, salinity stress reduced grain weight and number by affecting osmotic processes, causing ion toxicity or Na^+^ toxicity [51,52,53].

Saline water irrigation caused a significant reduction in growth parameters such as shoot length, fresh and dry weights of the shoot, leaf number per plant, and leaf area. In addition to the disturbance in physiological and metabolic processes, salinity stress reduced root development by reducing nutrient and water uptake through increasing soil osmotic potential [30]. Our results are consistent with the finding of Hussein et al. [54] who reported that salinity reduced stem, leaf, and whole plant dry weights in maize. In maize, salinity stress decreases the leaf area [55]; decreases biomass accumulation [54,56], stem and root length, and grain yield [9]. Leaf growth rate rapidly reduces under salinity stress due to a reduction in photosynthetic activity [56] and cell elongation [57]. The deleterious effect of salinity stress can happen from the combination of water stress, ion toxicities, ion imbalance, or a combination of all these factors [58]. Regarding root length, our study found no effect of salinity on the root length of maize; this seems to contradict the results reported by [55,59] in maize and sugar beet.

### 3.2. Effect of Phosphorus on Plant Growth and Yield

Phosphorus fertilization significantly increased dry matter and grain yield under both saline and non-saline conditions. However, the positive effect of phosphorus was more pronounced under saline conditions (Figure 2), indicating P application as a risk minimization strategy under medium and high salinity conditions. Consistent with our results, P fertilization improved the above and below-ground dry weight and yield of barley by increasing crop tolerance against salinity [30,60]. Phosphorus application mitigates partially the adverse effects of salinity on maize [51], mung bean [61], green bean [35], chickpea [62], and wheat [31]. The increased crop yield under high salinity irrigation water can be attributed to the role of P, which increased concentration and uptake of essential plant nutrients such as N, and decreased the concentration and uptake of toxic ions. The improvement of the yield of crops by P supply in saline conditions is justified by an increase in plant height, nitrogen, and phosphorus uptake [37].

Phosphorus application increased parameters of growth and yield attributes such as leaf number, plant height, root length, root and shoot weight, and leaf area of maize; this increase was more pronounced in the high levels of P (150 kg P_2_O_5_·ha^−1^) (Table 2). Its application under saline conditions reduced the adverse effect of salt stress in many crops [31,33,35,37,61,62,63]. This improvement in crop tolerance to salinity can be explained by the increase in P availability in soil, which indirectly improved absorption of Ca^2+^ and Mn^+^ or Mn^2+^ as a result of reduced sodium absorption by the plant [64]. Moreover, P application enhances the synergistic relationship between P and other beneficial elements like K^+^, Ca^2+^ and Mg^2+^ and toxic ions which balances osmotic equilibrium and enhances salt tolerance to some extent [38]. Similar to this result, under saline conditions, P fertilization decreased sodium content in barley [60] and maintained the equilibrium between beneficial (K^+^ and Ca^2+^) and toxic ion (Na^+^ and Cl^-^) in the roots and leaves of rice [27]. In addition, P application increases N uptake efficiency under salinity conditions in corn, mainly due to the improvement in P availability in the root zone and its uptake [63].

Application of P fertilizer at all salinity levels increased both above- and under-ground plant parts. Our results confirm the previous findings of Belouchrani et al. [37] who reported that increased P rate stabilized the plant height in saline soil. In a similar study, Tang et al. [65] found that Maize biomass improved significantly (by twice) when the plants were subjected to a high rate of P application under salinity compared to a low rate of P. P application under saline conditions and has positive effects on shoot and root biomass, root length, P concentration, and chlorophyll content in green beans [35,66]. Phosphorus reduces the harmful effects of salt by increasing the fresh weight of roots and the shoot lengths of barley [30]. 

## 4. Materials and Methods

### 4.1. Experimental Site

**Figure 6:**
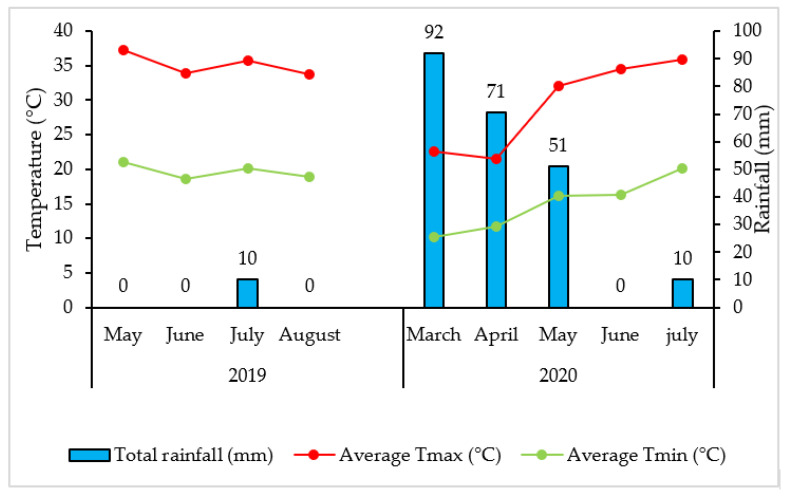
Temperature and rainfall during the experimental period in 2019 and 2020 in Tadla, Morocco.

This research was conducted between May 2019 to July 2020 at an experimental farm of the National Institute of Agronomic Research (INRA) in Tadla, Morocco (latitude = 32.2° N; Y = 6.31° W; altitude = 450 m). The soil of the experimental site is classified as Chromic Luvisols [67]. The climate is arid with high rainfall variability. The average annual pluviometry is 286 mm and the average temperature 18 °C, with the highest in August, which often exceeds 45 °C and the lowest in January which can range up to −3 °C (Figure 6).

### 4.2. Initial Soil and Water Characteristics

Before forage corn sowing. The sol analysis at two soil depths was performed following the protocol described by Jackson [68] (Table 2). The electrical conductivity of the soil was measured using the soil-saturated paste method with an EC meter (HI 9812, Hanna Instruments, Casablanca, Morocco). As the ECe values were relatively low (Table 3), the soil is non-saline [69].

Irrigation freshwater analysis is presented in Table 4 in terms of EC, pH cations and anions.

### 4.3. Experimental Design and Treatments

The field experiment was conducted over an area of about 1000 m^2^ in a split-plot design with three replicates applying four salinity level irrigation water and three phosphorus fertilization rates. The tested salinity levels were freshwater with an EC value of 0.7 dS·m^−1^ and three levels of saline water with an EC of 2, 4 and 6 dS·m^−1^. Salinity levels were achieved by adding salt (NaCl) to freshwater. The P fertilization rate consisted of 105 (the recommended rate used by farmers), 126 (plus 20%), and 150 kg P_2_O_5_·ha^−1^ (plus 40% of recommended rate). The area of each plot was 20 m^2^ (4 m × 5 m), and each consisted of six rows with a 75 cm interline. A 1 m distance was kept as buffer area between plots. For P, triple superphosphate (45% of P_2_O_5_) was applied, which is commonly used in the region. Phosphorus fertilizer was incorporated into the soil before sowing during the soil preparation which consisted of a deep ploughing using disc plough followed by a superficial ploughing using cover crop. P fertilizer was applied during the superficial soil preparation.

Other fertilizer requirements were applied equally for all treatments through fertigation with a drip irrigation system using integrated drippers with a discharge of 2 L·h^−1^ and a distance of 40 cm between drippers. Irrigation with saline water started 35 days after planting, and the crop was irrigated daily until harvest (20 August 2019 for the first season and 15 July 2020 for the second season). The amount of irrigation matched with the amount of potential evapotranspiration. Saline irrigation solutions were prepared in a separate tank of 1 m^3^ before each irrigation and irrigation water EC was monitored using EC meter. Forage corn was sown on 10 May 2019 and harvested on 20 August 2019 for the first season, while in the second season, it was sown on 15 April 2020 and harvested on 15 July 2020. The recommended seed rate of 100,000 seeds per ha was used with an inter-row distance equal to 75 cm and inter-plant distance equal to 13 cm. Thinning of plants (two per hole) was performed prior to the treatment application. The soil was supplemented with a total quantity of 162 kg of N·ha^−1^. About 42 kg^−1^ of N was applied at soil preparation using ammonium sulfate and 120 kg N·ha^−1^ using ammonium nitrate was applied through fertigation system during the growing period.

### 4.4. Observations

#### 4.4.1. Growth and Yield

The dry matter yield was measured at harvest using the whole plot area (20 m^2^) and then extrapolated to t·ha^−1^. Yield components, including root weight and length, plant height, shoot fresh weight and leaf number and area were determined using the values from six representative plants per plot. The sun-dried grain yield was measured at harvest using the half plot area (10 m^2^). The cobs harvested were sun-dried. Corn shelled and grain weight was measured one month after harvest of corn silage.

#### 4.4.2. Stomatal Conductance

Stomatal conductance was measured using the SC-1 Leaf Porometer (Decagon Devices, Inc., Pullman, WA, USA). It was determined between 10 am and 13 pm on the upper leaf surface well exposed to sunlight. One measurement per plant was carried out for four plants per plot once for each season.

### 4.5. Statistical Analysis

Statistical analysis was carried out using SPSS software version 17.0. A two-way analysis of variance (ANOVA) was used to assess the effects of both salinity and phosphorus rate monitored parameters. Before conducting the ANOVA, the normality of the data distribution was examined for dry matter and grain yield using the Shapiro–Wilk test. ANOVA combined over the season was performed as the season x treatment effect was not significant. The level of significance was set to *p* < 0.05. The treatment mean differences were analyzed using Tukey’s test (*p* ≤ 0.05).

Correlation and multivariate analysis were performed using the statistical programming language R 4.0.5. The “corrplot” package was used to display the Pearson correlation matrix values among variables, the level of significance was set to *p* < 0.05. Principal component analysis (PCA) was performed using “ggplot2”, “factoextra” and “FactoMineR” packages. 

## 5. Conclusions

Due to increased salinization problem in the world in general and specifically in Morocco, practices such fertilization could be a judicious solution to alleviate the adverse effect of salinity on crop productivity. The findings of our study reveal that phosphorus supply has a positive effect on growth parameters and the yield of forage corn under saline conditions. Thus, it improves the tolerance to salinity. In the light of results obtained, and in order to achieve a satisfactory yield, it is recommended to apply a phosphorus rate of 126 kg of P_2_O_5_ ha^−1^ under moderate salinity (4 dS·m^−1^) and 150 kg of P_2_O_5_ ha^−1^ under high salinity conditions (6 dS·m^−1^).

## Figures and Tables

**Table 1:** Results of ANOVA (analysis of variance) for all investigated parameters during 2019–2020.

Season	Salinity and P Rates	Number of Leaves	Plant Height	Root Length	Root Fresh Weight	Shoot Fresh Weight	Plant Dry Weight	Leaf Area	Stomatal Conductance	Dry Matter Yield	Grain Yield
2019	Salinity	0.04 *	0.01 **	0.03 *	0.10	0.05 *	0.04 *	0.01 **	0.02 *	0.01 **	0.03 *
	Phosphorus	0.20	0.20	0.01 **	0.04 *	0.03 *	0.01 **	0.20	0.04 *	0.04 *	0.2
	Interaction	0.40	0.60	0.07	0.15	0.02 *	0.04 *	0.40	0.2	0.2	0.3
2020	Salinity	0.03 *	0.02	0.01 **	0.30	0.04 *	0.03 *	0.02 *	0.03 *	0.03 *	0.04 *
	Phosphorus	0.18	0.40	0.02 *	0.01 **	0.02 *	0.01 **	0.40	0.01 *	0.04 *	0.01 **
	Interaction	0.30	0.30	0.12	0.70	0.09	0.01 **	0.60	0.13	0.005 **	0.015 *

* and ** indicate significance level at *p* = 0.05 and 0.01, respectively.

**Table 2:** Plant growth parameters under different irrigation water salinity and P rate. Values with the same letters under the same salinity level are statistically equal.

Season	Irrigation Water Salinity (dS·m^−1^)	P Rate (kg P_2_O_5_·ha^−1^)	Number of Leaves	Plant Height (cm)	Root Length (cm)	Root Fresh Weight (g·plant^−1^)	Shoot Fresh Weight (g·plant^−1^)	Plant Dry Weight (g)	Leaf Area (cm^2^·plant^−1^)
2019	0.7	105	13 ± 2 a	165 ± 15 b	22 ± 3 b	22 ± 4 b	279 ± 34 a	82 ± 39 b	25 ± 6 b
		126	14 ± 2 a	181 ± 8 a	21 ± 4 b	23 ± 8 b	282 ± 60 a	87 ± 61 b	25 ± 5 b
		150	14 ± 1 a	168 ± 14 b	22 ± 6 a	28 ± 11 a	284 ± 74 a	119 ± 43 a	26 ± 5 a
	2	105	11 ± 2 c	166 ± 18 a	20 ± 5 b	30 ± 9 b	271 ± 70 c	110 ± 27 a	24 ± 6 b
		126	12 ± 2 b	168 ± 14 a	22 ± 4 a	33 ± 12 a	284 ± 70 b	102 ± 45 a	25 ± 5 b
		150	12 ± 2 a	166 ± 18 a	21 ± 3 b	32 ± 9 a	292 ± 77 a	103 ± 58 a	28 ± 4 a
	4	105	12 ± 1 a	131 ± 10 c	22 ± 3 b	20 ± 7 b	209 ± 37 b	59 ± 35 c	19 ± 3 c
		126	12 ± 1 a	153 ± 12 a	25 ± 3 a	26 ± 8 a	236 ± 51 a	83 ± 28 b	22 ± 5 b
		150	13 ± 1 a	147 ± 12 b	25 ± 3 a	27 ± 8 a	241 ± 69 a	97 ± 43 a	22 ± 4 a
	6	105	13 ± 1 a	150 ± 11 b	24 ± 6 b	18 ± 3 c	200 ± 29 b	55 ± 31 c	19 ± 4 b
		126	13 ± 1 a	148 ± 6 b	24 ± 3 ab	19 ± 3 b	204 ± 28 b	76 ± 17 b	20 ± 2 b
		150	12 ± 1 a	154 ± 16 a	25 ± 4 a	22 ± 7 a	223 ± 67 a	81 ± 35 a	21 ± 5 a
2020	0.7	105	15 ± 1 a	154 ± 20 a	23 ± 4 a	22 ± 4 b	249 ± 74 b	97 ± 5 b	32 ± 15 a
		126	15 ± 4 a	146 ± 23 a	22 ± 5 a	24 ± 8 b	249 ± 87 b	95 ± 4 b	21 ± 7 a
		150	14 ± 3 a	137 ± 28 a	24 ± 8 a	25 ± 11 a	276 ± 80 a	113 ± 7 a	24 ± 8 a
	2	105	12 ± 3 a	153 ± 18 a	21 ± 5 b	32 ± 9 b	288 ± 84 a	116 ± 5 a	26 ± 7 a
		126	11 ± 1 a	156 ± 29 a	21 ± 4 b	34 ± 12 a	268 ± 100 a	114 ± 4 a	19 ± 8 a
		150	13 ± 4 a	163 ± 24 a	22 ± 4 a	33 ± 15 a	285 ± 111 a	118 ± 7 a	26 ± 9 a
	4	105	12 ± 4 b	134 ± 14 b	24 ± 3 b	22 ± 7 b	178 ± 44 b	74 ± 11 b	19 ± 4 b
		126	13 ± 4 a	159 ± 16 a	26 ± 3 a	26 ± 8 a	266 ± 65 a	106 ± 7 a	24 ± 5 a
		150	13 ± 2 a	152 ± 24 a	24 ± 4 a	26 ± 8 a	267 ± 105 a	107 ± 6 a	25 ± 8 a
	6	105	13 ± 1 a	148 ± 12 b	23 ± 6 b	19 ± 7 c	169 ± 40 c	78 ± 9 c	18 ± 4 c
		126	12 ± 2 a	152 ± 6 ab	25 ± 3 b	23 ± 3 a	222 ± 30 b	90 ± 4 b	21 ± 3 b
		150	12 ± 2 a	167 ± 24 a	25 ± 4 a	22 ± 7 b	251 ± 82 a	97 ± 4 a	22 ± 6 a

Small letters (a, b and c) indicate the statistically homogenous groups.

**Table 3:** Initial soil characteristics in the experimental site Tadla, 2019.

Soil Depth (cm)	Clay (%)	Silt (%)	Sand (%)	Soil pH	pH		EC (dS·m^−1^)	Organic Matter (%)	Total N (Kjeldahl) (g·kg^−1^)	P_2_O_5_ (Olsen) (mg·kg^−1^)	K_2_O (Acetate of Na) (mg·kg^−1^)
					Water	KCl					
0–20	28.1	52.8	19.1	7.92	8.24	7.36	0.1	1.45	2.34	43	459
20–40	43.1	18.7	38.2	8.09	8.38	7.24	0.22	0.59	3.44	22	405

**Table 4:** Irrigation freshwater chemical analysis.

EC (dS/m)	pH	Cations (meq/L)				Anions (meq/L)				
		Ca^2+^	Mg^2+^	Na^+^	K^+^	Cl^−^	SO_4_^2−^	CO_3_^2−^	HCO_3_^−^	NO_3_^−^
0.7	7.4	2.4	3.9	2.290	0.001	2.25	0.54	1.2	4.3	0.12452

## Data Availability

Data is contained within the article.
